# The Use of Three Long Non-Coding RNAs as Potential Prognostic Indicators of Astrocytoma

**DOI:** 10.1371/journal.pone.0135242

**Published:** 2015-08-07

**Authors:** Feng Zhi, Qiang Wang, Lian Xue, Naiyuan Shao, Rong Wang, Danni Deng, Suinuan Wang, Xiwei Xia, Yilin Yang

**Affiliations:** 1 Modern Medical Research Center, Third Affiliated Hospital of Soochow University, Changzhou, 213000, Jiangsu, China; 2 Department of Neurosurgery, Third Affiliated Hospital of Soochow University, Changzhou, 213000, Jiangsu, China; CSIR Institute of Genomics and Integrative Biology, INDIA

## Abstract

Long noncoding RNAs (lncRNAs) are pervasively transcribed and play a key role in tumorigenesis. The aim of the study was to determine the lncRNA expression profile in astrocytomas and to assess its potential clinical value. We performed a three-step analysis to establish the lncRNA profile for astrocytoma: a) the lncRNA expression was examined on 3 astrocytomas as well as 3 NATs (normal adjacent tissues) using the lncRNA microarray; b) the top-hits were validated in 40 astrocytomas (WHO grade II-IV) by quantitative real time-PCR (qRT-PCR); c) the hits with significant differences were re-evaluated using qRT-PCR in 90 astrocytomas. Finally, 7 lncRNAs were found to have a significantly different expression profile in astrocytoma samples compared to the NAT samples. Unsupervised clustering analysis further revealed the potential of the 7-lncRNA profile to differentiate between tumors and NAT samples. The upregulation of ENST00000545440 and NR_002809 was associated with advanced clinical stages of astrocytoma. Using Kaplan-Meier survival analysis, we showed that the low expression of BC002811 or XLOC_010967, or the high expression of NR_002809 was significantly associated with poor patient survival. Moreover, Cox proportional hazard regression analysis revealed that this prognostic impact was independent of other clinicopathological factors. Our results indicate that the lncRNA profile may be a potential prognostic biomarker for the prediction of post-surgical outcomes.

## Introduction

Astrocytomas are the most common primary malignant brain tumor in the central nervous system [1]. According to the 2007 World Health Organization (WHO) classification, astrocytomas can be categorized into 4 grades based on their histological and morphological features [2]. Despite new biological insights and therapeutic advances, the general prognosis for astrocytoma patients remains poor, particularly in patients with grade IV astrocytomas (glioblastoma multiforme, GBM), who have a median survival time of only 15 months [3]. A better understanding of the genetic and molecular disorders of the disease is the key to early diagnosis, appropriate treatment and improved prognosis of patients with astrocytoma.

Recently, long non-coding RNAs (lncRNAs) have attracted increasing scientific interest. LncRNAs are transcripts longer than 200 nucleotides that are not translated into proteins and are found in sense or antisense orientation to protein-coding genes, within introns of protein-coding genes or in intergenic regions of the genome [4–5]. A significant proportion of lncRNAs have intrinsic RNA-mediated functions in *trans*, while the majority of lncRNAs are thought to function in *cis* (through the act of their transcription) [6]. An increasing number of studies have suggested that lncRNAs are deregulated in different types of cancer and function as tumor suppressors or oncogenes [7–8]. Undoubtedly, lncRNAs have become new players in cancer pathogenesis after microRNAs, although the detailed mechanisms of most lncRNAs remain largely unknown.

Although the clinical stage of cancer is the primary predictor of survival for patients who have undergone surgery for most solid tumors, including astrocytoma, the predictions are not very accurate. Patients of the same stage with similar treatment may have very different clinical outcomes. Unique patterns of altered lncRNA expression may serve as novel molecular biomarkers for astrocytoma. Han et al. found that the lncRNA profile in GBM was significantly altered and may be involved in GBM pathogenesis [9]. Zhang et al. demonstrated that specific lncRNA expression profiles were correlated with different histological subtypes and malignancy grades in human glioma, and they identified a set of 6 lncRNAs that were significantly associated with overall survival in GBM patients [10–11]. Hackermuller et al. found that 126 known lncRNAs were differentially expressed between astrocytomas of grade I compared to the aggressive states grades III and IV [12]. Yan et al. found that 815 lncRNAs were differentially expressed between the GBM and normal brain groups [13]. Li et al. classified three molecular subtypes in glioma patients based on lncRNA expression profiles [14]. Although these studies established their own lncRNA signatures for astrocytoma, the results were from a small number of cases, or were only specific to GBM, or were from statistical analysis, or were not associated with prognosis.

In the present study, we investigated the lncRNA expression profile of human astrocytoma by comparing the expression levels of lncRNAs in WHO grade II-IV astrocytoma samples with that from normal adjacent tissue (NAT) samples using a high-throughput lncRNA microarray followed by quantitative real-time PCR (qRT-PCR). We observed a widespread variation in the levels of lncRNA expression during astrocytic tumorigenesis. Notably, the profile of seven specific lncRNAs exhibited great potential to differentiate astrocytomas from NAT samples. The upregulation of ENST00000545440 and NR_002809 was associated with advanced clinical stages of astrocytoma. Using the Kaplan-Meier survival analysis and univariate/multivariable statistical models, we showed that low expression levels of BC002811 or XLOC_010967 and high expression levels of NR_002809 were significantly associated with poor survival in astrocytoma patients. These results indicate that NR_002809, BC002811 and XLOC_010967 have the potential to serve as novel prognostic indicators of astrocytoma.

## Materials and Methods

### Study design, patients and control subjects

The present study included 130 patients who underwent surgical treatment for treat astrocytomas at the Third Affiliated Hospital of Soochow University between 2005 and 2013. The study was approved by the Research Ethics Board of the Third Affiliated Hospital of Soochow University, and written informed consent was obtained from each participant.

The astrocytoma cases were individuals with newly diagnosed, histologically confirmed primary astrocytomas. Histological subtypes were defined according to WHO criteria. There were no age, gender or cancer-grade restrictions on recruitment. The following inclusion criteria were used: (i) the absence of previous cancers or recurrent tumors, (ii) the absence of previous chemo- or radiotherapeutic treatment and (iii) the absence of synchronous multiple cancers. Sixty NAT samples were also analyzed and served as controls. The NAT samples were the same as those described in our previous work [15]. The NAT samples were the peritumoral brain tissues, from the brain tissue adjacent to the tumor and involved in edema, and were histologically confirmed normal brain tissues by at least three independent pathologists. The NAT samples are normal adjacent samples from tumor patients but not of those included as disease cases in the analysis. All tissue samples were stored in liquid nitrogen until the time of analysis. Clinical follow-up examinations were performed every 3 months during the first year after surgery, and every 6 months during the second year, followed by an annual exam thereafter until death. The time to the event was measured from the time of surgery to death or to the last recorded follow-up visit for the included patients.

A multiphase, case-control study was designed to identify lncRNAs as potential markers for astrocytomas. In the initial biomarker screening stage, an lncRNA microarray was performed on 3 astrocytoma samples (1 WHO grade II, 1 WHO grade III, and 1 WHO grade IV) and 3 NAT samples to identify lncRNA differences between astrocytomas and controls. Subsequently, sequential validation was performed using qRT-PCR to refine the number of lncRNAs included in the astrocytoma signature. All 130 samples included in the confirmation stage were randomly separated into training (40 astrocytoma samples and 20 NAT samples) and validation (90 astrocytoma samples and 40 NAT samples) sets prior to analysis. The demographic and clinical features of the patients are listed in Table 1.

**Table 1:** Summary of the demographic and clinical features of the astrocytoma and NAT samples.

	Training Set			Validation set			*p-*value ^c^
Variable	Astrocytoma (n = 40)	NAT (n = 20)	*p-*value ^a^	Astrocytoma (n = 90)	NAT (n = 40)	*p-*value ^b^	
**Average age (years)**	45.3 ± 13.3	44.1 ± 11.8	0.535 ^d^	46.2 ± 15.0	44.8 ± 12.6	0.427 ^d^	0.372 ^d^
**Age (years)**			0.927 ^e^			0.977 ^e^	0.571 ^e^
≤ 46	18	10		47	21		
> 46	22	10		43	19		
**Sex**			0.854 ^e^			0.860 ^e^	0.837 ^e^
Male	23	11		48	22		
Female	17	9		42	18		
**WHO grade**							
Diffuse astrocytoma (WHO grade II)	15			29			
Anaplastic astrocytoma (WHO grade III)	13			31			
Glioblastoma multiforme (GBM, WHO grade IV)	12			30			
**Follow-up**							
Alive	30			57			0.270 ^e^
Dead	10			33			
Mean survival time (months)	42.3 ± 17.3			45.7 ± 8.6			

^a^ Astrocytoma samples from training set versus control samples from training set.

^b^ Astrocytoma samples from validation set versus control samples from validation set.

^c^ Astrocytoma samples from training set versus astrocytoma samples from validation set.

^d^ Student's t-test.

^e^ Two-sided λ^2^ test.

### LncRNA microarray

Total RNA was first extracted from 3 tumor samples and 3 NAT samples using Trizol reagent (Invitrogen) according to manufacturer’s protocol. The Agilent human lncRNA + mRNA Array v2.0 was used in this study. The microarray experiment and data analysis were performed by CapitalBio, Beijing, PR China. A detailed version of the procedure is included in the S1 Method.

### Selection of a suitable target for normalisation

Two algorithms, geNorm [16] and NormFinder [17] were used to assess the expression stability of putative normaliser genes. The geNorm algorithm calculates the average expression stability (M value) of a gene by using pairwise comparisons with a cut-off value of 0.15, ranking putative reference genes according to the similarity of expression profiles across a sample set. NormFinder is a model-based approach that determines the expression stability of candidate reference genes according to their group origin. This approach determines the inter- and intra-group variation and combines both results in a stability value for each gene. According to NormFinder, genes with the lowest stability will be ranked highest.

### Quantification of lncRNAs by qRT-PCR analysis

For qRT-PCR, the reverse transcription reactions were carried out with Reverse Transcriptase (SuperScript III, Invitrogen) according to the manufacturer’s instructions. Approximately 2μg total RNA was added to each reaction. The TaqMan gene expression assay (Invitrogen) was performed on an ABI 7500 system in a 20μl reaction. All the primers and probes were designed and produced by Invitrogen. The reactions were incubated at 95°C for 5 min, followed by 40 cycles of 95°C for 15 s, and 60° for 60 s. All quantitative PCR reactions were performed in triplicate. Each lncRNA in each sample was repeated by qRT-PCR for at least 3 times. The Ct value of each candidate lncRNA was then normalized to the expression value of GAPDH. Relative expression levels of the lncRNAs were calculated using the 2^-△Ct^ method.

### Statistical analysis

Statistical comparison of the demographic features between the astrocytoma and NAT samples, or between the astrocytoma samples from the training and validation sets, was performed by Student’s t-test or two-sided λ^2^ test. The differences were considered statistically significant when p < 0.05. We constructed the receiver operating characteristic (ROC) curve and calculated the area under the ROC curve (AUC) to evaluate the potential power of the lncRNA signature for astrocytoma. Risk score analysis was performed to evaluate the associations between the expression levels of the lncRNAs and astrocytoma. The risk score of each lncRNA, denoted as *s*, was set to 1 if the expression level was greater than the upper 95% reference interval for the corresponding lncRNA level in the controls; otherwise, it was set to 0. A risk score function (RSF) to predict astrocytoma risk was defined according to a linear combination of the expression level for each lncRNA. For example, the RSF for sample *i* using the information from *n* lncRNAs was $rsf_{i}=\sum_{j=1}^{n}W_{j}\cdot s_{ij}$. In the above equation, *s*_*ij*_ is the risk score for lncRNA *j* on sample *i*, and *W*_*j*_ is the weight of the risk score of lncRNA *j*. To determine the Ws, *n* univariate logistic regression models were fitted using the disease status with each of the risk scores. The regression coefficient of each risk score was used as the weight to indicate the contribution of each lncRNA to the RSF. Moreover, we identified the lncRNAs with expression levels significantly related to patient survival. The survival curves were estimated using the Kaplan-Meier method in SPSS 13.0, and the resulting curves were compared using a log-rank test. We also computed a level of statistical significance for each lncRNA based on a univariate Cox proportional hazard regression model in SPSS 13.0. The joint effect of covariables was examined using a multivariate Cox proportional hazard regression model in SPSS 13.0. The differences were considered statistically significant when p < 0.05.

## Results

### Demographic and clinical features of astrocytoma patients

A total of 130 histologically confirmed astrocytoma patients from the Third Affiliated Hospital of Soochow University, ranging from WHO grade II to grade IV, were enrolled in the present study. Additionally, 60 NAT samples were analyzed and served as controls. These samples were randomly assigned to a training set (40 astrocytoma samples and 20 NAT samples) or to a validation set (90 astrocytoma samples and 40 NAT samples). The demographic and clinical features of the astrocytoma and NAT samples are listed in Table 1. There was no significant difference in the demographic factors between the patient samples and the controls, or between the patient samples from the training set and the validation set. Of the 40 tumor samples in the training set, 15, 13, and 12 samples were diagnosed as diffuse astrocytoma, anaplastic astrocytoma or glioblastoma multiforme, respectively. Of the 90 tumor samples in the validation set, 29, 31, and 30 samples were characterized as diffuse astrocytoma, anaplastic astrocytoma or glioblastoma multiforme, respectively. There was no significant difference in tumor grade between the patient samples in the training set and the validation set. All 130 astrocytoma patients received follow-up examinations. Among the 40 patients in the training set, the median follow-up time was 42.3 months (range 13–102 months). During the follow-up period, 10 patients (25.0%) died from the disease. Of the 90 patients in the validation set, the median follow-up was 45.7 months (range 14–110 months), and 33 patients (36.7%) died during this period.

### Selection of candidate lncRNAs for astrocytoma using microarray analysis

To select candidate lncRNA biomarkers for astrocytomas, we first performed an initial lncRNA screening of 3 astrocytoma samples and 3 NAT samples by lncRNA microarray. The results revealed that the lncRNA expression profiles varied between the astrocytomas and the NAT samples. Among the lncRNAs detected, 3806 lncRNAs with a fold change > 2 or < 0.5 and a q value < 0.05 were found to have significantly different expression levels in astrocytoma samples compared to NAT samples. Of those, 1196 were downregulated and 2610 were upregulated. These lncRNAs were listed in S1 Table. Hierarchical clustering of these lncRNAs clearly separated the astrocytoma samples from the NAT samples (S1 Fig). We next narrowed down the list of lncRNAs to be used as astrocytoma lncRNA profile. The following criteria were used to select the lncRNA for further analysis based our experience: 1) the raw gProcessed Signal > 500, 2) retained all probes that did not overlap protein coding transcripts. 3) the lncRNA length > 500 kb and < 2500 kb. The antisense transcripts were retained. Too small sequences bring the risk of losing some regulatory sites, and reduce the signal. Too large sequences enhance the noise and reduce the significance. Consequently, 59 lncRNAs (31 being downregulated and 28 upregulated) that met the inclusion criteria were chosen (S2 Table). The microarray data has been deposited in NCBI Gene Expression Omnibus (GEO) database under the accession number GSE58276.

### Selection of a suitable target for normalization

Proper normalization is a critical aspect of quantitative gene expression analysis. An algorithm known as geNorm was used to assess the expression stability of 4 putative normalizer genes (GAPDH, β-actin, 18s rRNA and 28s rRNA). The geNorm analysis clearly showed that GAPDH was highly consistent in their expression levels across 40 astrocytoma tissue samples and 20 NAT samples in the training set (Fig A in S2 Fig). GAPDH was statistically superior to other most commonly used reference RNAs. When the gene expression stability was estimated independently using the NormFinder software, the result was essentially the same as that from geNorm. The NormFinder algorithm selected GAPDH as the optimal reference gene for normalization (Fig B in S2 Fig). In the subsequent experiments, ubiquitously expressed GAPDH was used as a normalization control for the qRT-PCR assay.

### Validation of the microarray results by qRT-PCR

The 59 candidate lncRNAs were individually assayed by qRT-PCR in the 130 astrocytoma samples and 60 NAT samples, including the samples used in the microarray, to validate their differential expression. Only the lncRNAs with a mean fold change > 2 or < 0.5 and a p-value < 0.05 were selected from the training set for further validation. LncRNAs were excluded from further analysis when their expression levels were not significantly altered, the assays were not linear, the detection rates were <50%, or the Ct values were higher than 35 in the qRT-PCR assay. Based on these parameters, our analysis generated a total of 9 lncRNAs that were differentially expressed between astrocytomas and NAT samples (Table 2). To verify the accuracy and specificity of these lncRNAs and to refine the number of lncRNAs to be used as the astrocytoma signature, we further assessed the 9 lncRNAs in the validation sample set. The lncRNAs were considered significantly altered only when they exhibited a mean fold-change > 2 or < 0.5 relative to the controls, a p-value < 0.05 and a parallel trend of variation between the training set and the validation set. Our analysis ultimately generated a list of 7 lncRNAs that were differentially expressed in astrocytomas in comparison to the NAT samples (Table 2). Among these lncRNAs, ENST00000244906, ENST00000545440, NR_002809 and ENST00000436616 were shown to be upregulated by a factor greater than twofold, whereas 3 lncRNAs, XLOC_010967, BC002811 and ASO1937, were shown to be downregulated by a factor greater than twofold.

**Table 2:** Validation by qRT-PCR of differentially expressed lncRNAs in astrocytoma samples compared to NAT samples.

LncRNA	Training set		Validation set		Training + Validation		Result
	Mean fold (tumor/NAT)	p-value	Mean fold (tumor/NAT)	p-value	Mean fold (tumor/NAT)	p-value	
ENST00000244906	5.05	3.61 × 10^−4^	3.19	4.74 × 10^−2^	3.399	4.06 × 10^−3^	significant
ENST00000545440	2.45	6.82 × 10^−3^	3.72	1.64 × 10^−6^	2.610	2.61 × 10^−6^	significant
NR_002809	2.21	1.30 × 10^−2^	2.47	3.44 × 10^−2^	2.201	1.24 × 10^−2^	significant
ENST00000436616	2.74	4.71 × 10^−2^	2.35	1.31 × 10^−2^	2.050	1.00 × 10^−2^	significant
XLOC_010967	0.40	3.62 × 10^−2^	0.42	5.18 × 10^−4^	0.474	7.81 × 10^−4^	significant
BC002811	0.31	5.37 × 10^−6^	0.48	2.39 × 10^−4^	0.429	7.11 × 10^−8^	significant
ASO1937	0.19	1.50 × 10^−3^	0.34	4.88 × 10^−3^	0.290	3.63 × 10^−5^	significant
ENST00000424474	2.09	4.87 × 10^−3^	1.25	3.64 × 10^−1^			not significant
uc002hng.1	0.48	1.47 × 10^−5^	0.88	6.74 × 10^−2^			not significant

**Fig 1:**
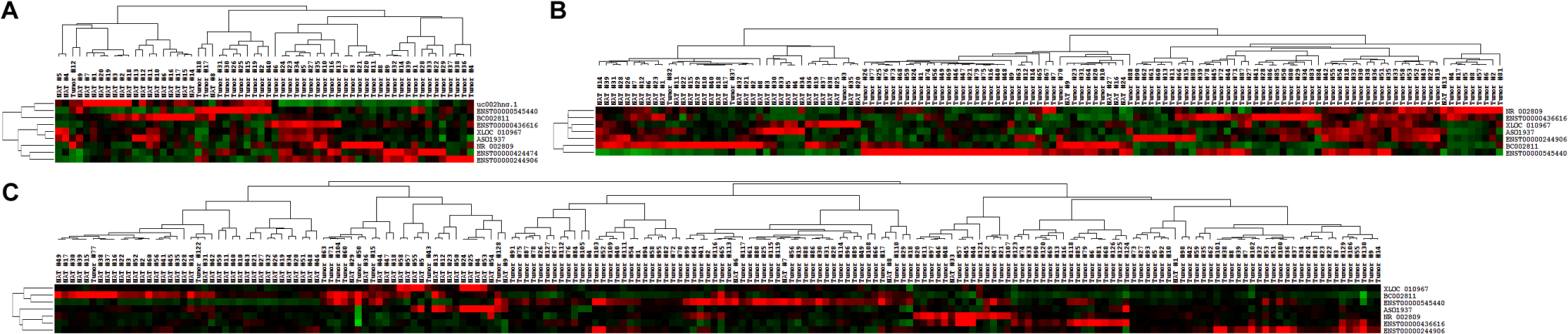
Cluster analysis of differentially expressed lncRNAs between astrocytomas and NAT samples. The lncRNA expression levels, as measured by qRT-PCR, were normalized, mean-centered, clustered, and plotted as a heat map for the training set (A), the validation set (B), and all samples (C). The dendrogram generated by the cluster analysis shows a clear separation of the astrocytoma and the NAT samples based on the 9 or 7 lncRNA profiles.

The differential expression of lncRNAs between astrocytoma samples and NAT samples was further characterized by an unsupervised clustering analysis that was blind to the clinical annotations. The dendrogram generated by the cluster analysis showed a clear separation of the astrocytoma samples from the NAT samples based on their respective lncRNA profiles (Fig 1). Of the 40 astrocytoma samples and 20 NAT samples from the training set, only 1 astrocytoma sample and 1 NAT sample were incorrectly classified (Fig 1A). In the validation set, 90 astrocytoma samples and 40 NAT samples were also clearly separated into two main classes, with 3 astrocytoma samples and 5 NAT samples incorrectly classified (Fig 1B). Finally, a similar result was obtained when we combined the samples from the training set and the validation set, with 10 astrocytoma samples and 5 NAT samples incorrectly classified among the 130 astrocytoma samples and 60 NAT samples (Fig 1C).

Among the mRNAs detected, 3547 mRNAs with a fold change > 2 or < 0.5 and a p value < 0.05 were found to have significantly different expression levels in astrocytoma samples compared to NAT samples. Of those, 1959 were upregulated and 1588 were downregulated. Among the mRNAs with the raw gProcessed Signal > 500, two of the 5 most upregulated mRNAs (Tenascin-C, Aquaporin-1) and two of the 5 most downregulated mRNAs (HAPLN4, PPP2R2C) were validated in the training set (40 astrocytomas and 20 NAT samples). As shown in S3 Fig, Tenascin-C, and Aquaporin-1 were significantly upregulated in astrocytoma tissues compared with NAT samples, while HAPLN4 and PPP2R2C were significantly downregulated. These results were coincided with prior studies [18–21].

To assess the power of the lncRNA signature, we used a risk score formula to calculate the risk score for patient samples and control samples in the training set. The samples were ranked according to their risk score and then divided into a high-risk group, which represented the predicted astrocytoma cases, and a low-risk group, which represented the predicted control individuals. The ROC (receiver operating characteristic) curve is a graphical plot that illustrates the performance of a binary classifier system as its discrimination threshold is varied. The frequency table and the ROC curve were then used to evaluate the power of the 9-lncRNA panel. The AUC for the combined 9 lncRNAs was 0.9992 (95% CI, 0.9990 to 1.0003) for the astrocytomas and controls (S4 Fig).

### Correlation of lncRNA expression with demographic and clinical factors

**Fig 2:**
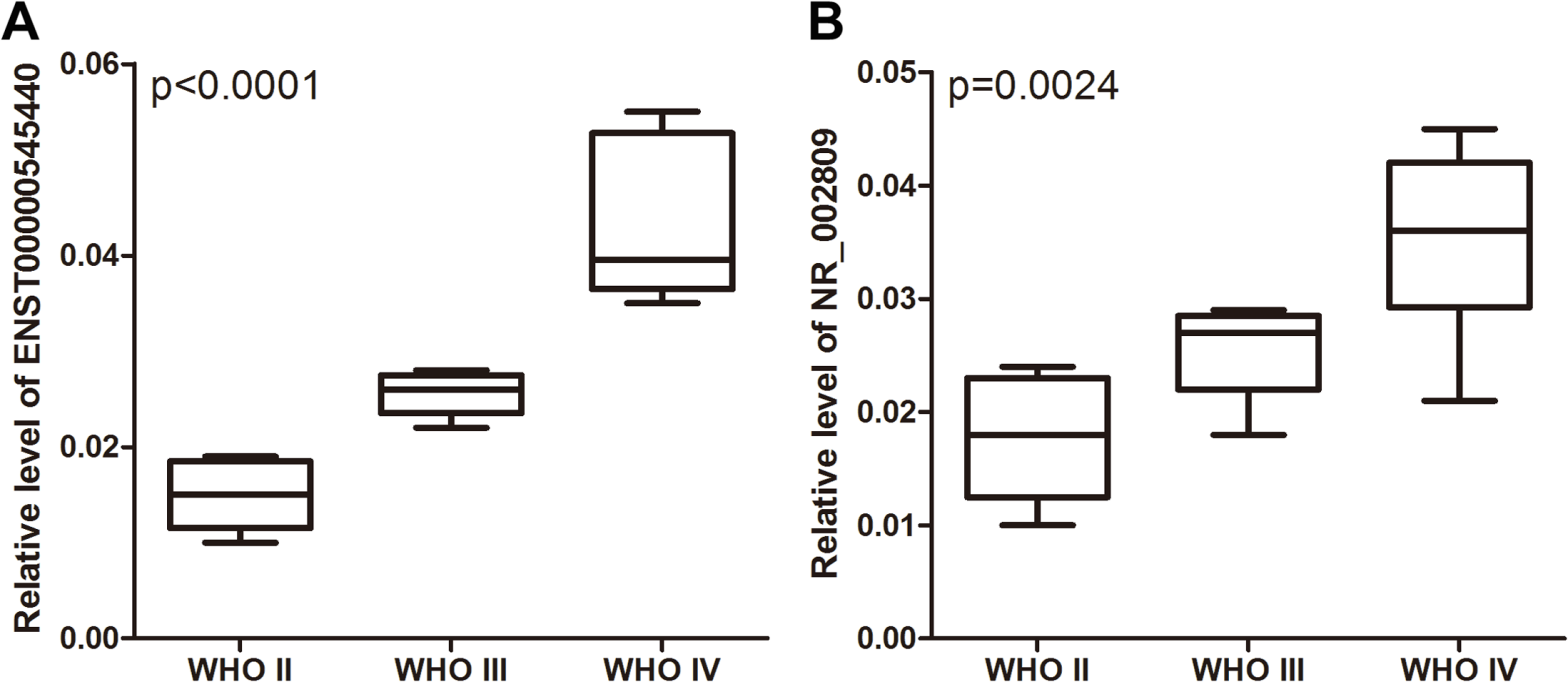
The levels of lncRNA expression in the astrocytoma patients stratified by tumor grade. The relative expression levels of ENST00000545440 and NR_002809 in each group are shown.

We subsequently investigated whether lncRNA expression levels represented specific molecular signatures for subsets of astrocytomas. The expression levels of the 7 lncRNAs in the astrocytoma samples were stratified using 3 types of clinicopathological parameters (sex, age, and WHO grade). We assessed the relationship between these clinical features and the lncRNA expression levels. No lncRNAs were found to be differentially expressed when the astrocytoma samples were stratified by age or sex. However, 2 lncRNAs were found to be differentially expressed when the samples were stratified according to tumor grade. As shown in Fig 2, the expression of ENST00000545440 and NR_002809 increased from WHO grade II to WHO grade IV astrocytomas. This result suggests that the upregulation of ENST00000545440 and NR_002809 is associated with advanced clinical stages of astrocytomas.

### Correlation between lncRNA expression profiles and survival of astrocytoma patients

**Fig 3:**
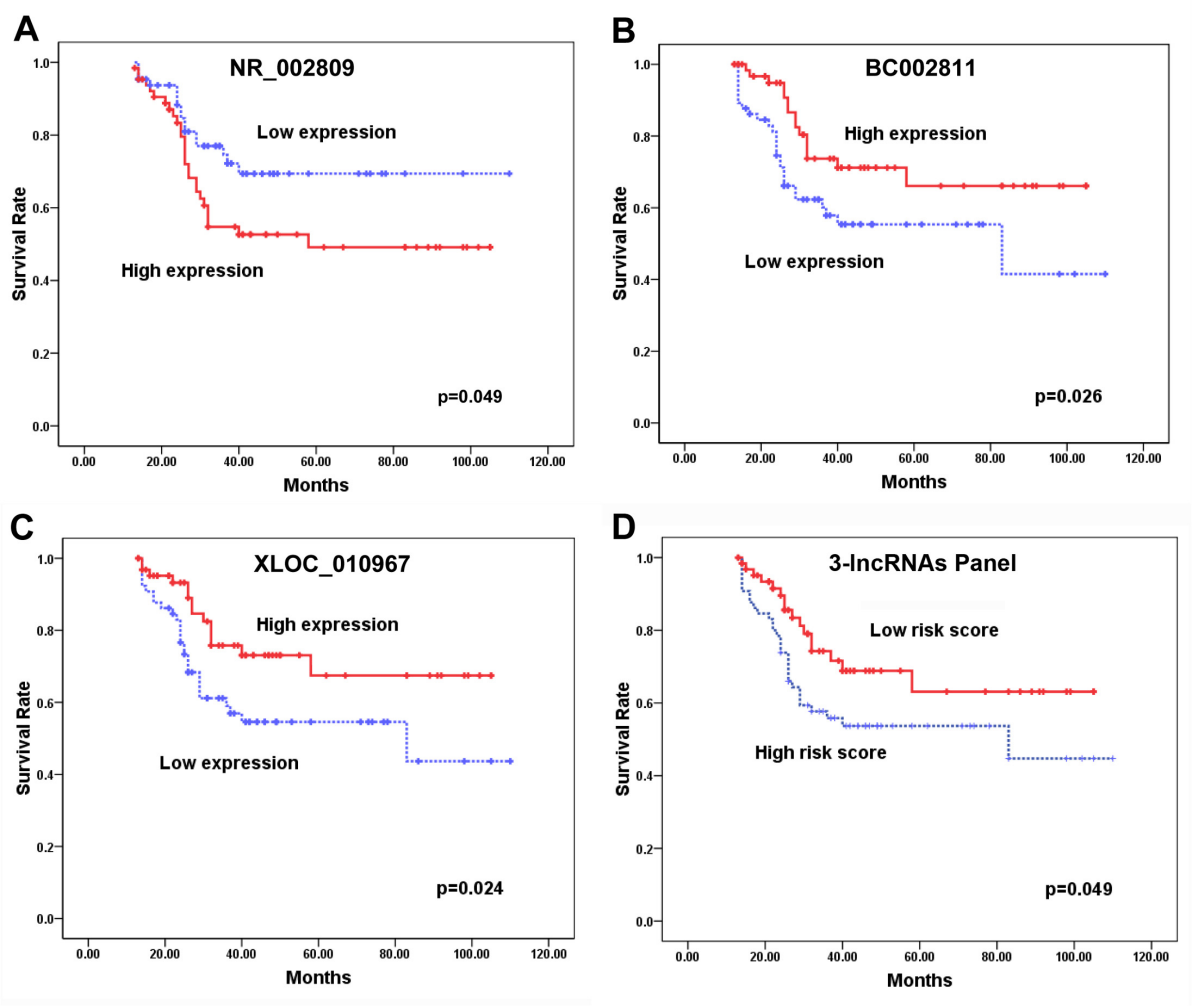
The relationship between the lncRNA expression levels and the survival time of the astrocytoma patients. (A) Kaplan-Meier survival analysis of the astrocytoma patients stratified according to the expression level of NR_002809. (B) Kaplan-Meier survival analysis of the astrocytoma patients stratified according to the expression level of BC002811. (C) Kaplan-Meier survival analysis of the astrocytoma patients stratified according to the expression level of XLOC_010967. (D) Kaplan-Meier survival analysis of the astrocytoma patients according to the 3-lncRNA signature stratified by risk score.

We next investigated the correlation between the lncRNA expression profiles and patient survival using the prospective follow-up data collected from the 130 astrocytoma patients. Due to the observation that 7 lncRNAs were differentially expressed between the astrocytoma patients and the controls, these lncRNAs were used for the survival analysis. The expression levels of these 7 lncRNAs in the astrocytoma samples were first stratified by the median value; then, the survival of the patients with high lncRNA expression levels (≥ median) was compared with the outcomes for patients with low lncRNA expression levels (< median), as determined by Kaplan-Meier survival analysis. We observed a marginally significant poorer survival rate in astrocytoma patients who expressed high levels of NR_002809 (p = 0.049, Fig 3A), and low levels of BC002811 (p = 0.026, Fig 3B) and XLOC_010967 (p = 0.024, Fig 3C). The results suggested that following tumor resection, the expression of NR_002809, BC002811, and XLOC_010967 may have a prognostic value for astrocytoma patients. To further evaluate the prognostic value of the 3-lncRNA profiling system, Kaplan-Meier survival analysis was used to compare the patients with high-risk and low-risk scores. The risk score was calculated using information from the 3 lncRNAs according to the equation described in the methods for each patient. The risk scores of these patients were stratified by the median value. The high risk score was the risk score ≥median, while the low risk score was the risk score<median. The patients with high-risk scores had a poorer survival rate than those with low-risk scores (p = 0.049) (Fig 3D).

Subsequently, a univariate Cox proportional hazard regression model was performed to determine the influence of lncRNA expression, as well as clinicopathological characteristics (gender, age and WHO grade), on patient survival. WHO grade II was designated as the low pathological grade, and WHO III-IV was designated as the high pathological grade. This univariate analysis indicated that age, WHO grade and the expression levels of NR_002809, BC002811, and XLOC_010967 were significantly related to survival (hazard ratio >2 and p-value <0.05 were considered to be statistically significant), whereas gender was not (Table 3). To adjust for the potentially confounding effects of univariate modeling of age, gender or WHO grade, a multivariate Cox proportional hazard regression analysis using all of these clinicopathological factors was performed. The multivariate analysis revealed that old age, high NR_002809 expression level and low BC002811 and XLOC_010967 expression levels were independently associated with decreased survival (Table 3). These results suggest that BC002811 expression levels are an important prognostic predictor, independent of other clinicopathological factors.

**Table 3:** Survival analysis of astrocytoma patients in relation to the clinicopathological characteristics and lncRNA expression.

Variable	Subset	Hazard ratio (95% CI)	p-value
Univariate analysis			
Gender	Female/Male	1.032 (0.563–1.894)	0.918
Age	Age≥46/Age<46	5.088 (2.420–10.696)	< 0.0001
WHO	High grade/Low grade	3.167 (1.404–7.470)	0.005
NR_002809	High/Low	2.962 (1.523–5.869)	0.042
BC002811	Low/High	2.061 (1.041–3.965)	0.037
XLOC_010967	Low/High	2.086 (1.097–3.968)	0.025
Multivariate analysis			
Gender	Female/Male	0.749 (0.395–1.422)	0.377
Age	Age≥46/Age<46	6.163 (2.722–13.702)	< 0.0001
WHO	High grade/Low grade	2.328 (0.966–5.442)	0.051
NR_002809	High/Low	3.022 (1.129–8.087)	0.028
BC002811	Low/High	4.573 (1.863–11.228)	0.001
XLOC_010967	Low/High	2.782 (1.122–6.893)	0.027

## Discussion

In the present study, we examined the lncRNA profiles of astrocytoma samples and NAT samples and identified a unique astrocytoma signature composed of 7 differentially expressed lncRNAs. Unsupervised clustering analysis revealed a clear separation of astrocytoma samples from NAT samples, indicating that these 7 lncRNAs may represent an astrocytoma lncRNA ‘‘fingerprint.” The upregulation of ENST00000545440 and NR_002809 was associated with advanced clinical stages of astrocytoma. Moreover, the low expression of BC002811 and XLOC_010967, or high expression of NR_002809 was significantly associated with poor patient survival.

An increasing number of studies have suggested deregulation of lncRNAs in various cancers. In the present study, we provide a ''proof-of-principle'' approach to identify a particular disease-specific lncRNA profile. This approach included microarray analysis as an initial screening followed by multiple qRT-PCR validation sets at the individual level. Employing this approach, we identified a unique expression profile for astrocytoma. In the present study, we identified a unique lncRNA signature of astrocytoma comprising 7 differentially expressed lncRNAs. However, these lncRNAs were different from those found in previous studies. Indeed, we investigate the lncRNA expression profiles in a sample set including 40 NAT samples and 130 astrocytoma samples across grades II-IV, while the lncRNA signatures established by other studies were only from a limited patient cohort, or were only specific to GBM, or were not associated with prognosis, or were just from statistical analysis by literature screen. Though no single lncRNA was found in common, our study on astrocytoma lncRNAs was more comprehensive and more systematic. The reason for limited overlap between our study and other previous studies may be due to the differences in study design, race, sample size and methodology.

Like protein-coding genes and microRNAs, lncRNAs can function as oncogenes or tumor suppressors during cancer progression. The mechanisms through which lncRNAs contribute to the regulatory networks that underpin cancer development are diverse. The functions of lncRNAs are intimately linked to their gene structures. Thus, understanding the gene structure for these molecules is essential to determining how they function. Though many investigators have suggested the presence and importance of structural elements within lncRNAs, lncRNA structure remains poorly understood. LncRNAs act through a variety of mechanisms such as remodeling of chromatin, transcriptional co-activation or co-repression, protein inhibition, and posttranscriptional modifiers or decoy elements [22]. Consequently, altered expression of lncRNAs can lead to changes in the expression profiles of various target genes involved in different aspects of cell homeostasis [23]. A further investigation of the roles and mechanisms of lncRNAs in cancer will provide novel lncRNA-based strategies for the treatment of human cancers. Some lncRNAs have been reported as oncogenes. HOTAIR promotes glioblastoma cell cycle progression in an EZH2 dependent manner, while its reduction induced colony formation suppression, cell cycle G0/G1 arrest, and orthotopic tumor growth inhibition [24–25]. H19 promotes glioma cell invasion by deriving miR-675 [26]. POU3F3 promotes cell viability and proliferation in glioma cells [27]. Some lncRNAs are reported as tumor suppressors. ROR inhibits the cell proliferation and reduces the CD133 expression rate and glioma stem sphere-forming ability [28]. CASC2 suppresses cell proliferation, migration, and invasion, and promotes cell apoptosis in human gliomas by miR-21 [29]. TSLC1-AS1 inhibits cell proliferation, migration and invasion in glioma cells [30]. ADAMTS9-AS2 is regulated by DNMT1 and inhibits the migration of glioma cells [31]. However, up to now, there have been no direct reports regarding the roles of the identified lncRNAs in our study in cancer development.

Seeking novel molecular biomarkers of malignancy is important and helpful for clinical diagnosis and management. The discovery that lncRNAs are key regulators in cancer transformation and progression leads to intriguing possibilities of application for diagnostics and therapeutics. The use of noncoding RNAs in diagnostics has intrinsic advantages over protein-coding RNAs. Although lncRNAs may require post-transcriptional modifications or protein interactions to function, because the mature product is the functional end-product, measurement of its expression directly represents the levels of the active molecule. Many lncRNAs are expressed in a tissue- and cancer-type restricted manner and have already been shown to be useful as prognostic markers. HOTAIR was strongly increased in primary tumors and metastases of breast cancer patients, with expression levels positively correlated with a poor survival rate [32]. MVIH was found to be overexpressed in hepatocellular carcinoma and was significantly associated with decreased recurrence-free survival and overall survival [33]. H19 was underexpressed in intratumoral hepatocellular carcinoma tissues (T) compared to peritumoral tissues (L), and a low T/L ratio of H19 was associated with shorter disease-free survival and can be used to predict poor prognosis [34]. In glioma, MALAT1 was shown to be upregulated and served as an independent prognostic parameter for patient survival [35]. In our study, we found that the low expression of BC002811 and XLOC_010967 and the high expression of NR_002809 were significantly associated with poor patient survival. However, the reason that these three lncRNAs appear to have a prognostic impact on the survival has yet to be elucidated. There have been no reports about the roles of these three lncRNAs in astrocytoma development. Nevertheless, additional studies to investigate how the altered expression of these lncRNAs contribute to the development and/or progression of astrocytomas would improve our understanding of the molecular basis of this tumor and may ultimately lead to novel therapeutic interventions, as well as a prognostic tool for this disease.

In conclusion, our study identified an lncRNA signature of astrocytoma and presents the first assessment of the impact of lncRNAs on the survival of astrocytoma patients. Further validation studies in prospective cohorts and in cohorts from different institutions are needed to test the prognostic power of the signature before it is applied in a clinical setting. This observation should initiate further research to elucidate the functional effects of these lncRNAs, which will improve our knowledge regarding the role that these novel biomarkers play in carcinogenesis and will elucidate their potential as therapeutic agents.

## Supporting Information

S1 Fig Cluster analysis of lncRNAs that were differentially expressed between 3 astrocytomas and 3 NAT samples based on lncRNA microarray (T = tumor, N = NAT). (TIF)

S2 Fig The stability of the selected reference genes by qRT-PCR assay. The expression levels of 4 selected candidates were measured using qRT-PCR from astrocytoma (n = 40) and NAT (n = 20) samples. The CT values were averaged, and the standard deviation was calculated. A) Identification of the optimal number of reference genes for accurate normalization using geNorm. B) Identification of the most stable reference genes using NormFinder.
(TIF)

S3 Fig The relative expression of Tenascin-C, Aquaporin-1, HAPLN4 and PPP2R2C in astrocytomas and NAT samples. For comparison, the expression levels of these 4 genes in NAT samples were arbitrarily set at 1.
(TIF)

S4 Fig Receiver operating characteristic (ROC) curve for the 9-lncRNA profile to distinguish the astrocytoma samples from the control samples. (TIF)

S1 Method The procedure of lncRNA microarray experiment and data analysis. (DOC)

S1 Table All the potential lncRNAs detected from microarray. (XLS)

S2 Table The candidate lncRNAs selected for qRT-PCR. (XLS)

## References

[1] Wen PY, Kesari S. Malignant gliomas in adults. N Engl J Med. 2008;359(5): 492–507. 10.1056/NEJMra0708126 18669428

[2] Louis DN, Ohgaki H, Wiestler OD, Cavenee WK, Burger PC, Jouvet A, et al The 2007 WHO classification of tumours of the central nervous system. Acta Neuropathol. 2007;114(2): 97–109. 17618441 10.1007/s00401-007-0243-4 PMC1929165

[3] Gabayan AJ, Green SB, Sanan A, Jenrette J, Schultz C, Papagikos M, et al GliaSite brachytherapy for treatment of recurrent malignant gliomas: a retrospective multi-institutional analysis. Neurosurgery. 2006;58(4): 701–709; discussion 701–709. 16575334 10.1227/01.NEU.0000194836.07848.69

[4] Birney E, Stamatoyannopoulos JA, Dutta A, Guigo R, Gingeras TR, Margulies EH, et al Identification and analysis of functional elements in 1% of the human genome by the ENCODE pilot project. Nature. 2007;447(7146): 799–816. 17571346 10.1038/nature05874 PMC2212820

[5] Clark MB, Johnston RL, Inostroza-Ponta M, Fox AH, Fortini E, Moscato P, et al Genome-wide analysis of long noncoding RNA stability. Genome Res. 2012;22(5): 885–898. 10.1101/gr.131037.111 22406755 PMC3337434

[6] Guttman M, Donaghey J, Carey BW, Garber M, Grenier JK, Munson G, et al lincRNAs act in the circuitry controlling pluripotency and differentiation. Nature. 2011;477(7364): 295–300. 10.1038/nature10398 21874018 PMC3175327

[7] Prensner JR, Chinnaiyan AM. The emergence of lncRNAs in cancer biology. Cancer Discov. 2011;1(5): 391–407. 10.1158/2159-8290.CD-11-0209 22096659 PMC3215093

[8] Qi P, Du X. The long non-coding RNAs, a new cancer diagnostic and therapeutic gold mine. Mod Pathol. 2013;26(2): 155–165. 10.1038/modpathol.2012.160 22996375

[9] Han L, Zhang K, Shi Z, Zhang J, Zhu J, Zhu S, et al LncRNA pro fi le of glioblastoma reveals the potential role of lncRNAs in contributing to glioblastoma pathogenesis. Int J Oncol. 2012;40(6): 2004–2012. 10.3892/ijo.2012.1413 22446686

[10] Zhang XQ, Sun S, Lam KF, Kiang KM, Pu JK, Ho AS, et al A long non-coding RNA signature in glioblastoma multiforme predicts survival. Neurobiol Dis. 2013;58: 123–131. 10.1016/j.nbd.2013.05.011 23726844

[11] Zhang X, Sun S, Pu JK, Tsang AC, Lee D, Man VO, et al Long non-coding RNA expression profiles predict clinical phenotypes in glioma. Neurobiol Dis. 2012;48(1): 1–8. 10.1016/j.nbd.2012.06.004 22709987

[12] Hackermuller J, Reiche K, Otto C, Hosler N, Blumert C, Brocke-Heidrich K, et al Cell cycle, oncogenic and tumor suppressor pathways regulate numerous long and macro non-protein-coding RNAs. Genome Biol. 2014;15(3): R48 10.1186/gb-2014-15-3-r48 24594072 PMC4054595

[13] Yan Y, Zhang L, Jiang Y, Xu T, Mei Q, Wang H, et al LncRNA and mRNA interaction study based on transcriptome profiles reveals potential core genes in the pathogenesis of human glioblastoma multiforme. J Cancer Res Clin Oncol. 2014. 10.1007/s00432-014-1861-6 PMC11824101 25378224

[14] Li R, Qian J, Wang YY, Zhang JX, You YP. Long noncoding RNA profiles reveal three molecular subtypes in glioma. CNS Neurosci Ther. 2014;20(4): 339–343. 10.1111/cns.12220 24393335 PMC6493123

[15] Zhi F, Chen X, Wang S, Xia X, Shi Y, Guan W, et al The use of hsa-miR-21, hsa-miR-181b and hsa-miR-106a as prognostic indicators of astrocytoma. Eur J Cancer. 2010;46(9): 1640–1649. 10.1016/j.ejca.2010.02.003 20219352

[16] Vandesompele J, De Preter K, Pattyn F, Poppe B, Van Roy N, De Paepe A, et al Accurate normalization of real-time quantitative RT-PCR data by geometric averaging of multiple internal control genes. Genome Biol. 2002;3(7): RESEARCH0034 12184808 10.1186/gb-2002-3-7-research0034 PMC126239

[17] Andersen CL, Jensen JL, Orntoft TF. Normalization of real-time quantitative reverse transcription-PCR data: a model-based variance estimation approach to identify genes suited for normalization, applied to bladder and colon cancer data sets. Cancer Res. 2004;64(15): 5245–5250. 15289330 10.1158/0008-5472.CAN-04-0496

[18] Sivasankaran B, Degen M, Ghaffari A, Hegi ME, Hamou MF, Ionescu MC, et al Tenascin-C is a novel RBPJkappa-induced target gene for Notch signaling in gliomas. Cancer Res. 2009;69(2): 458–465. 10.1158/0008-5472.CAN-08-2610 19147558

[19] El Hindy N, Bankfalvi A, Herring A, Adamzik M, Lambertz N, Zhu Y, et al Correlation of aquaporin-1 water channel protein expression with tumor angiogenesis in human astrocytoma. Anticancer Res. 2013;33(2): 609–613. 23393355

[20] Sim H, Hu B, Viapiano MS. Reduced expression of the hyaluronan and proteoglycan link proteins in malignant gliomas. J Biol Chem. 2009;284(39): 26547–26556. 10.1074/jbc.M109.013185 19633295 PMC2785343

[21] Fan YL, Chen L, Wang J, Yao Q, Wan JQ. Over expression of PPP2R2C inhibits human glioma cells growth through the suppression of mTOR pathway. FEBS Lett. 2013;587(24): 3892–3897. 10.1016/j.febslet.2013.09.029 24126060

[22] Fitzgerald KA, Caffrey DR. Long noncoding RNAs in innate and adaptive immunity. Curr Opin Immunol. 2014;26: 140–146. 10.1016/j.coi.2013.12.001 24556411 PMC3932021

[23] Cheetham SW, Gruhl F, Mattick JS, Dinger ME. Long noncoding RNAs and the genetics of cancer. Br J Cancer. 2013;108(12): 2419–2425. 10.1038/bjc.2013.233 23660942 PMC3694235

[24] Zhang K, Sun X, Zhou X, Han L, Chen L, Shi Z, et al Long non-coding RNA HOTAIR promotes glioblastoma cell cycle progression in an EZH2 dependent manner. Oncotarget. 2015;6(1): 537–546. 25428914 10.18632/oncotarget.2681 PMC4381613

[25] Zhang JX, Han L, Bao ZS, Wang YY, Chen LY, Yan W, et al HOTAIR, a cell cycle-associated long noncoding RNA and a strong predictor of survival, is preferentially expressed in classical and mesenchymal glioma. Neuro Oncol. 2013;15(12): 1595–1603. 10.1093/neuonc/not131 24203894 PMC3829598

[26] Shi Y, Wang Y, Luan W, Wang P, Tao T, Zhang J, et al Long non-coding RNA H19 promotes glioma cell invasion by deriving miR-675. PLoS One. 2014;9(1): e86295 10.1371/journal.pone.0086295 24466011 PMC3900504

[27] Guo H, Wu L, Yang Q, Ye M, Zhu X. Functional linc-POU3F3 is overexpressed and contributes to tumorigenesis in glioma. Gene. 2015;554(1): 114–119. 10.1016/j.gene.2014.10.038 25445282

[28] Feng S, Yao J, Chen Y, Geng P, Zhang H, Ma X, et al Expression and Functional Role of Reprogramming-Related Long Noncoding RNA (lincRNA-ROR) in Glioma. J Mol Neurosci. 2015. 10.1007/s12031-014-0488-z 25651893

[29] Wang P, Liu YH, Yao YL, Li Z, Li ZQ, Ma J, et al Long non-coding RNA CASC2 suppresses malignancy in human gliomas by miR-21. Cell Signal. 2015;27(2): 275–282. 10.1016/j.cellsig.2014.11.011 25446261

[30] Qin X, Yao J, Geng P, Fu X, Xue J, Zhang Z. LncRNA TSLC1-AS1 is a novel tumor suppressor in glioma. Int J Clin Exp Pathol. 2014;7(6): 3065–3072. 25031725 PMC4097230

[31] Yao J, Zhou B, Zhang J, Geng P, Liu K, Zhu Y, et al A new tumor suppressor LncRNA ADAMTS9-AS2 is regulated by DNMT1 and inhibits migration of glioma cells. Tumour Biol. 2014;35(8): 7935–7944. 10.1007/s13277-014-1949-2 24833086

[32] Gupta RA, Shah N, Wang KC, Kim J, Horlings HM, Wong DJ, et al Long non-coding RNA HOTAIR reprograms chromatin state to promote cancer metastasis. Nature. 2010;464(7291): 1071–1076. 10.1038/nature08975 20393566 PMC3049919

[33] Yuan SX, Yang F, Yang Y, Tao QF, Zhang J, Huang G, et al Long noncoding RNA associated with microvascular invasion in hepatocellular carcinoma promotes angiogenesis and serves as a predictor for hepatocellular carcinoma patients' poor recurrence-free survival after hepatectomy. Hepatology. 2012;56(6): 2231–2241. 10.1002/hep.25895 22706893

[34] Zhang L, Yang F, Yuan JH, Yuan SX, Zhou WP, Huo XS, et al Epigenetic activation of the MiR-200 family contributes to H19-mediated metastasis suppression in hepatocellular carcinoma. Carcinogenesis. 2013;34(3): 577–586. 10.1093/carcin/bgs381 23222811

[35] Ma KX, Wang HJ, Li XR, Li T, Su G, Yang P, et al Long noncoding RNA MALAT1 associates with the malignant status and poor prognosis in glioma. Tumour Biol. 2015. 10.1007/s13277-014-2969-7 25613066

