# Supplementary figures and images for: The Use of Three Long Non-Coding RNAs as Potential Prognostic Indicators of Astrocytoma
Source: PLoS One. 2015 Aug 7;10(8):e0135242. doi: 10.1371/journal.pone.0135242 (PMC4529097; doi:10.1371/journal.pone.0135242)

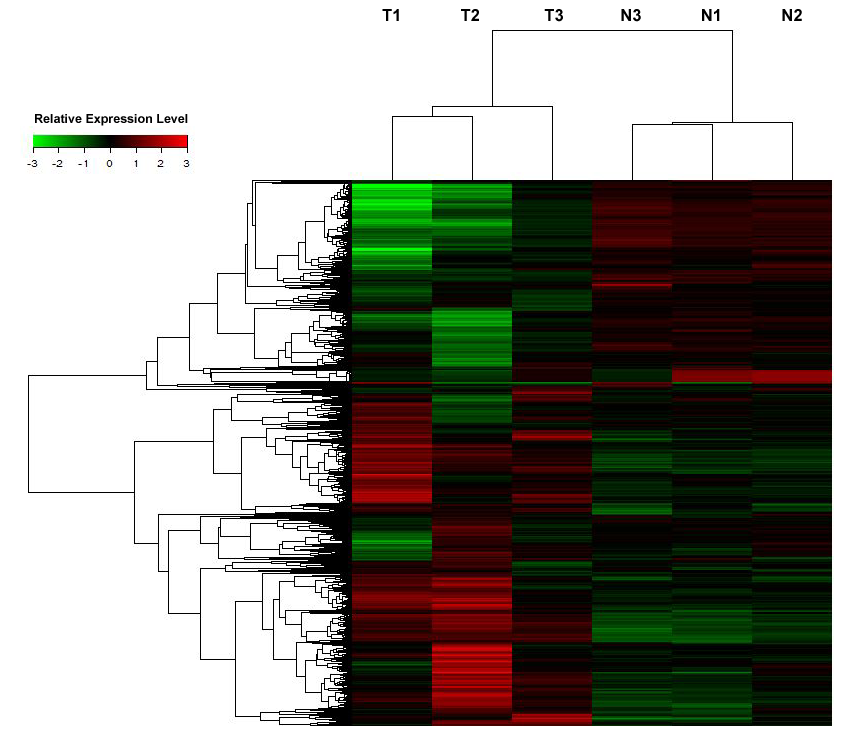

Supplement: S1 Fig — (TIF) [file pone.0135242.s001.tif]

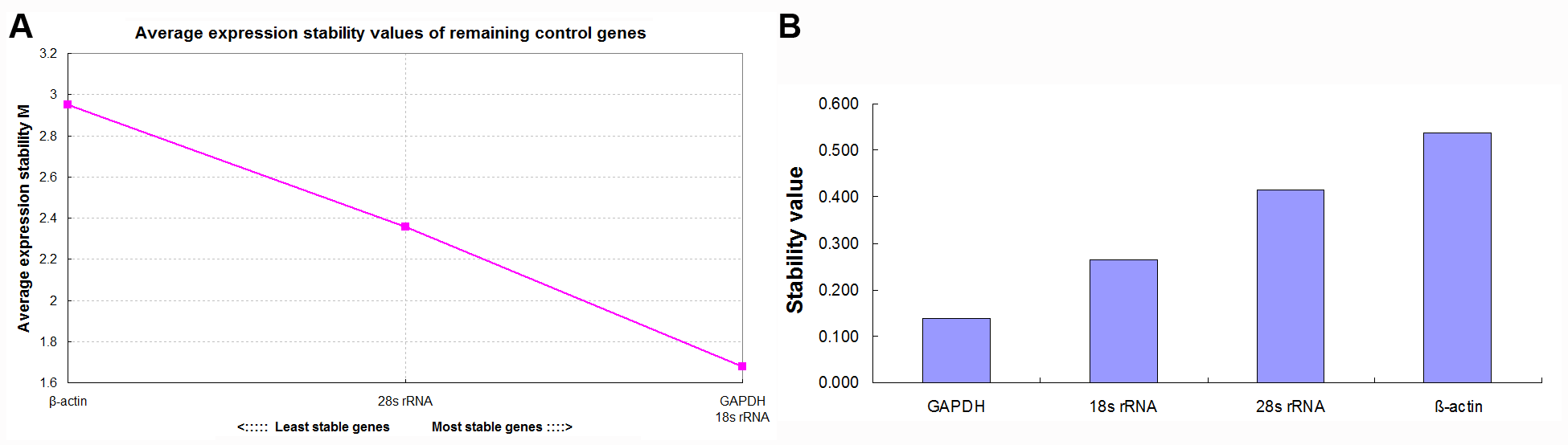

Supplement: S2 Fig — The expression levels of 4 selected candidates were measured using qRT-PCR from astrocytoma (n = 40) and NAT (n = 20) samples. The CT values were averaged, and the standard deviation was calculated. A) Identification of the optimal number of reference genes for accurate normalization using geNorm. B) Identification of the most stable reference genes using NormFinder. (TIF) [file pone.0135242.s002.tif]

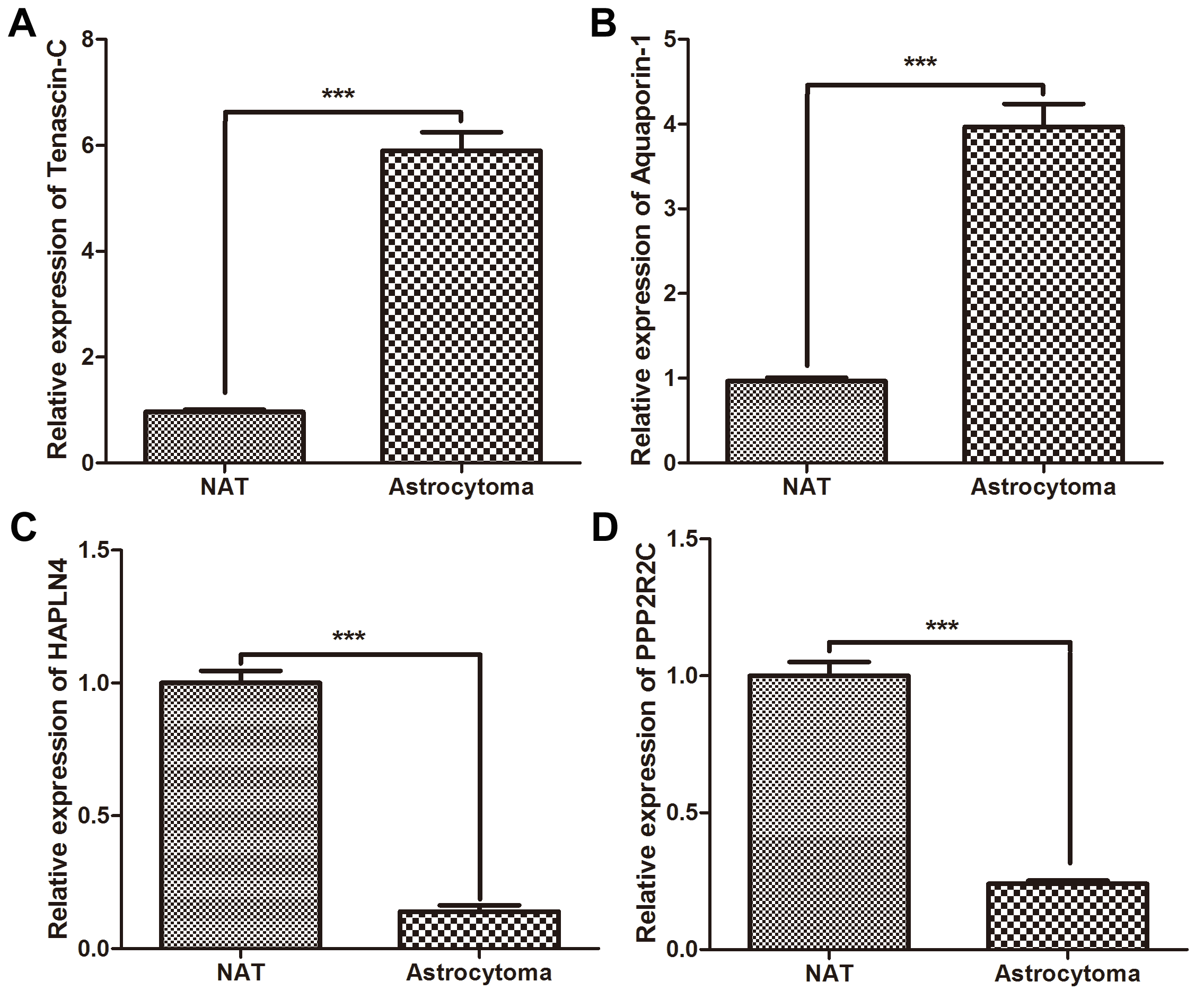

Supplement: S3 Fig — For comparison, the expression levels of these 4 genes in NAT samples were arbitrarily set at 1. (TIF) [file pone.0135242.s003.tif]

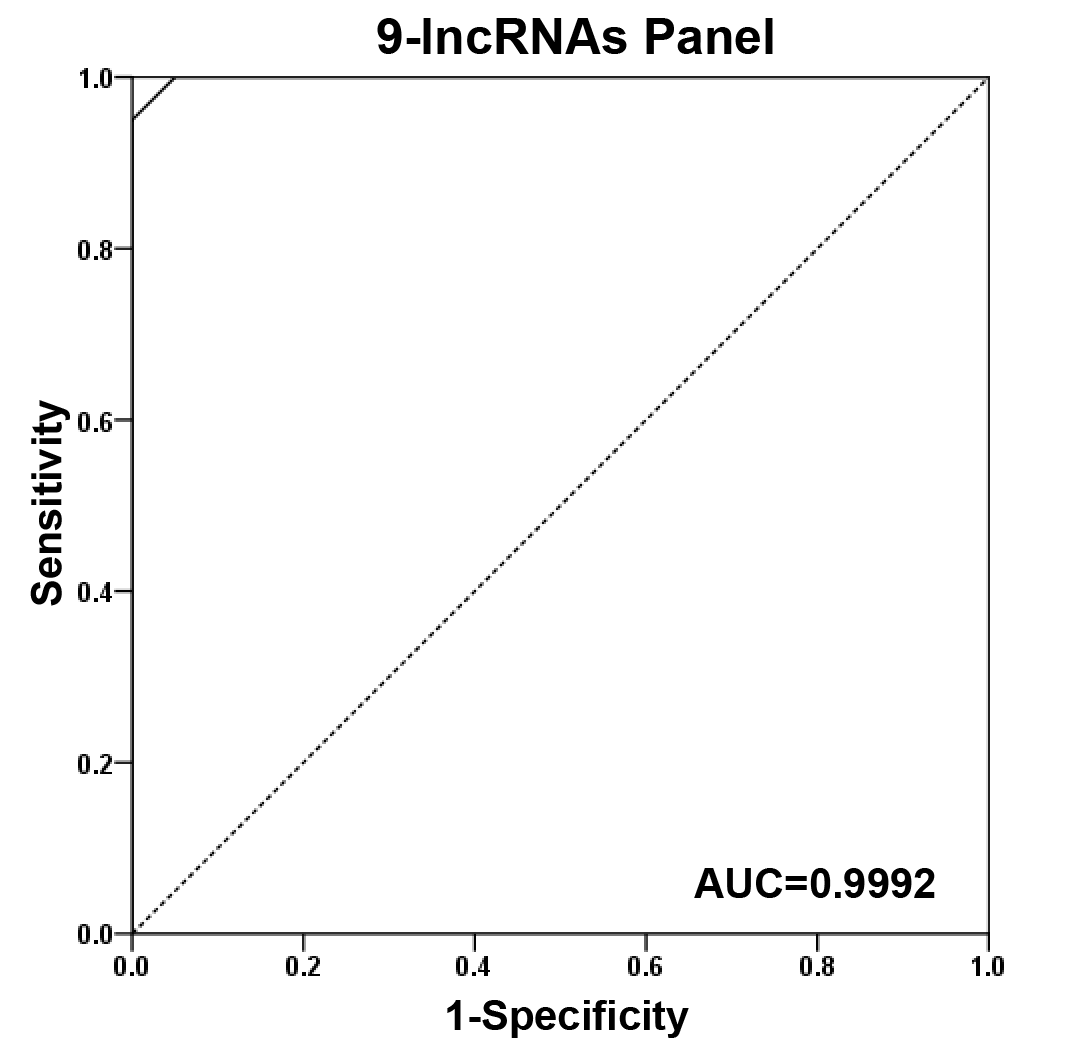

Supplement: S4 Fig — (TIF) [file pone.0135242.s004.tif]
