# Supplementary material for: The Use of Three Long Non-Coding RNAs as Potential Prognostic Indicators of Astrocytoma
Source: PLoS One. 2015 Aug 7;10(8):e0135242. doi: 10.1371/journal.pone.0135242 (PMC4529097; doi:10.1371/journal.pone.0135242)
Supplement: S1 Method — (DOC) [file pone.0135242.s005.doc]

**Method**

**Fabrication of DNA microarray**

The Agilent human lncRNA + mRNA Array v2.0 was designed with four identical arrays per slide (4 × 180K format), with each array containing probes interrogating approximately 39000 human lncRNAs and approximately 32000 human mRNAs. Those lncRNA and mRNA target sequences were merged from multiple databases, 4765 from RefSeq, 12754 from ENSEMBL, 8195 lincRNA from the John Rinn lab [1], 1289 from NRED (ncRNA Expression Database), 17203 from H-InvDB, 2975 from ENCODE, 529 from CombinedLit, 1053 from Antisense ncRNA pipeline, 407 Hox ncRNAs, 481 UCRs, and 848 from the Chen Ruisheng lab (Institute of Biophysics, Chinese Academy of Science). Each RNA was detected by probes repeat for 2 times. The array also contains 4974 Agilent control probes.

**RNA extraction, labeling and hybridization**

Total RNA containing small RNA was extracted using the Trizol reagent (Invitrogen) according to the manufacturer’s protocol. The purity and concentration of RNA were determined from OD260/280 readings using a spectrophotometer (NanoDrop ND-1000). RNA integrity was determined by 1 % formaldehyde denaturing gel electrophoresis. RNA integrity was further determined by capillary electrophoresis using the RNA 6000 Nano Lab-on-a-Chip kit and the Bioanalyzer 2100 (Agilent Technologies, Santa Clara, CA, USA). Only RNA extracts with RNA integrity values > 6 were included in further analysis.

**RNA amplification, labeling and hybridization**

cDNA labeled with a fluorescent dye (Cy5 and Cy3-dCTP) was produced by Eberwine’s linear RNA amplification method and subsequent enzymatic reaction. This procedure has been previously described [2], and the procedure has been improved by using a CapitalBio cRNA Amplification and Labeling Kit (CapitalBio) to produce higher yields of labeled cDNA. Briefly, double-stranded cDNAs (containing the T7 RNA polymerase promoter sequence) were synthesized from 1 μg total RNA using the CbcScript reverse transcriptase with cDNA synthesis system according to the manufacturer’s protocol (Capitalbio) with the T7 Oligo (dT) and T7 Oligo (dN). After completion of double-stranded cDNA (dsDNA) synthesis using DNA polymerase and RNase H, the dsDNA products were purified using a PCR NucleoSpin Extract II Kit (MN) and eluted with 30 μL elution buffer. The eluted double-stranded cDNA products were vacuum evaporated to 16 μL and subjected to 40 μL in vitro transcription reactions at 37°C for 14 hr using a T7 Enzyme Mix. The amplified cRNA was purified using an RNA Clean-up Kit (MN). Klenow enzyme labeling strategy was adopted after reverse transcription using CbcScript II reverse transcriptase. Briefly, 2 μg amplified RNA was mixed with 4 μg random nanomer, denatured at 65°C for 5 min, and cooled on ice. Then, 5 μL of 4 × first-strand buffer, 2 μL of 0.1 M DTT, and 1.5 μL CbcScript II reverse transcriptase were added. The mixtures were incubated at 25°C for 10 min, then at 37°C for 90 min. The cDNA products were purified using a PCR NucleoSpin Extract II Kit (MN) and vacuum evaporated to 14 μL. The cDNA was mixed with 4 μg random nanomer, heated to 95°C for 3 min, and snap cooled on ice for 5 min. Then, 5 μL Klenow buffer, dNTP, and Cy5-dCTP or Cy3-dCTP (GE Healthcare) were added to final concentrations of 240 μM dATP, 240 μM dGTP, 240 μM dTTP, 120 μM dCTP, and 40 μM Cy-dCTP. 1.2 μL Klenow enzyme was then added, and the reaction was performed at 37 ℃ for 90 min. Labeled cDNA was purified with a PCR NucleoSpin Extract II Kit (MN) and resuspended in elution buffer. Controls and test samples labeled with Cy5-dCTP and Cy3-dCTP were dissolved in 80 μL hybridization solution containing 3 × SSC, 0.2 % SDS, 5 × Denhardt’s solution and 25 % formamide. DNA in hybridization solution was denatured at 95°C for 3 min prior to loading onto a microarray. Arrays were hybridized in a Agilent Hybridization Oven overnight at a rotation speed of 20 rpm and a temperature of 42°C, after which they were washed with two consecutive solutions (0.2 % SDS, 2 × SSC at 42°C for 5 min, and 0.2 × SSC for 5 min at room temperature).

**Microarray imaging and data analysis**

The lncRNA + mRNA array data were analyzed for data summarization, normalization and quality control by using GeneSpring software V11.5 (Agilent). To select the differentially expressed genes, we used threshold values of ≥ 2 and ≤ -2-fold change and a Benjamini-Hochberg corrected p value of 0.05. The data were log2 transformed and median centered by gene using the Adjust Data function of CLUSTER 3.0 software, and then further analyzed with hierarchical clustering with average linkage. Finally, we performed tree visualization using Java Treeview (Stanford University School of Medicine, Stanford, CA, USA).

**References**

1. Orom UA, Derrien T, Beringer M, Gumireddy K, Gardini A, Bussotti G, et al. Long noncoding RNAs with enhancer-like function in human cells. Cell. 2010;143(1): 46-58.

2. Patterson TA, Lobenhofer EK, Fulmer-Smentek SB, Collins PJ, Chu TM, Bao W, et al. Performance comparison of one-color and two-color platforms within the MicroArray Quality Control (MAQC) project. Nat Biotechnol. 2006;24(9): 1140-1150.
